# Relationships between aggression, sensation seeking, brain stiffness, and head impact exposure: Implications for head impact prevention in ice hockey

**DOI:** 10.1002/brb3.2627

**Published:** 2022-05-27

**Authors:** Melissa S. DiFabio, Daniel R. Smith, Katherine M. Breedlove, Thomas A. Buckley, Curtis L. Johnson

**Affiliations:** ^1^ Department of Biomedical Engineering University of Delaware Newark Delaware USA; ^2^ Department of Child and Adolescent Psychiatry, Psychosomatics, and Psychotherapy Ludwig‐Maximillians‐Universität München Munich Germany; ^3^ Center for Clinical Spectroscopy Brigham and Women's Hospital Boston Massachusetts USA; ^4^ Department of Radiology Harvard Medical School Boston Massachusetts USA; ^5^ Department of Kinesiology and Applied Physiology University of Delaware Newark Delaware USA

**Keywords:** athletic training, injury management, neuroscience, sports medicine, sports psychology

## Abstract

**Objectives:**

The objectives of this study were to (1) examine the relationship between the number of head impacts sustained in a season of men's collegiate club ice hockey and behavioral traits of aggression and sensation seeking, and (2) explore the neural correlates of these behaviors using neuroimaging.

**Design:**

Retrospective cohort study.

**Methods:**

Participants (*n* = 18) completed baseline surveys to quantify self‐reported aggression and sensation‐seeking tendencies. Aggression related to playing style was quantified through penalty minutes accrued during a season. Participants wore head impact sensors throughout a season to quantify the number of head impacts sustained. Participants (*n* = 15) also completed baseline anatomical and magnetic elastography neuroimaging scans to measure brain volumetric and viscoelastic properties. Pearson correlation analyses were performed to examine relationships between (1) impacts, aggression, and sensation seeking, and (2) impacts, aggression, and sensation seeking and brain volume, stiffness, and damping ratio, as an exploratory analysis.

**Results:**

Number of head impacts sustained was significantly related to the number of penalty minutes accrued, normalized to number of games played (*r* = .62, *p* < .01). Our secondary, exploratory analysis revealed that number of impacts, sensation seeking, and aggression were related to stiffness or damping ratio of the thalamus, amygdala, hippocampus, and frontal cortex, but not volume.

**Conclusions:**

A more aggressive playing style was related to an increased number of head impacts sustained, which may provide evidence for future studies of head impact prevention. Further, magnetic resonance elastography may aid to monitor behavior or head impact exposure. Researchers should continue to examine this relationship and consider targeting behavioral modification programs of aggression to decrease head impact exposure in ice hockey.

## Graphical abstract

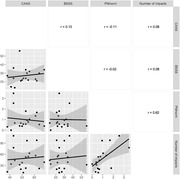

## 1 INTRODUCTION

There has been growing concern regarding the health‐related consequences of the cumulative effect of repetitive head impact exposure (HIE) from sports participation, as athletes of collision sports can sustain hundreds of head impacts in a single season, and thousands over the course of a career (Mainwaring et al., 2018). The consequences associated with greater HIE include changes in brain structure and function, increased risk of concussion, and may even serve as a catalyst to later life neural diseases and disorders (Mainwaring et al., 2018; McKee et al., 2009; Montenigro et al., 2017). Therefore, reducing HIE may be critical to increasing athlete safety and long‐term health.

Several interventions to reduce HIE have been studied to date, with most large‐scale studies targeting exogenous variables such as when or how often contact can occur, or altering existing equipment (Emery et al., 2019; Stemper et al., 2020; Swartz et al., 2019). However, these interventions have not been consistently successful in reducing HIE, perhaps because they do not address athlete's specific behaviors. Behavior modification programs encompass athlete‐specific strategies to effectively change the way athletes play in order to reduce HIE, though they have only been studied on a smaller scale (Champagne et al., 2019; Combs et al., 2019; Swartz et al., 2019). To date, successful behavior modification programs have included interventions such as education through watching personalized film or receiving individual education by coaches about tackling techniques (Champagne et al., 2019). It is still unknown how athletes’ personality traits may influence their HIE, though certain traits have been implicated in affecting the risk of sustaining sports‐related concussion. However, modification of such traits has potential to be targeted through psychoeducation‐based behavior modification programs; thus, understanding the relationships between these traits and HIE could also serve as a tool for identifying those who may be at risk for greater HIE and related neurological sequalae.

One such trait to consider is sports aggression, which can be defined as “any intentional behavior, not recognized as legal within the official rules of conduct, directed towards an opponent, official, team‐mate or spectator who is motivated to avoid such behavior” (Tenenbaum et al., 1997). Existing research has described ice hockey athletes as among the most aggressive in relation to athletes of other sports, and has defined penalty minutes (PM) accrued as a marker of aggression within the hockey community (Pedersen, 2007). Players who accrue more PM are considered more aggressive and may be at greater risk for contact‐related injury, including concussion (Agel et al., 2007; Cusimano et al., 2013; Emery & Meeuwisse, 2006). In fact, aggressive body checking has been identified as the primary act leading to concussions in hockey and accounts for the majority of “aggressive” PM assessed by referees (Bushman & Wells, 1998; Carré & McCormick, 2008; Emery et al., 2011; Gee & Leith, 2007). Based on the likelihood that accrual of more PM coincides with increased body contact, it is possible that more aggressive players, as measured by PM, may be more likely to sustain greater HIE.

Another behavioral trait closely related to aggression is sensation seeking, which is defined as “the need for varied, novel, and complex sensations and experiences, and the willingness to take physical and social risks for the sake of such experience” (Zuckerman, 1982). Interestingly, no studies have yet examined the role of sensation‐seeking tendencies on HIE, but a recent study found that college athletes who reported higher levels of sensation‐seeking tendencies at baseline were more likely to have a history of concussion and be more likely to sustain an in‐season concussion (Liebel et al., 2020). As greater HIE has been identified as a potential risk factor for concussion, it is possible that athletes with more HIE may also exhibit greater sensation‐seeking tendencies (Beckwith et al., 2013).

Although there seems to be an association between these behaviors and increased risk of physical contact or even head injury, no studies have directly studied how individuals’ perceived aggression or sensation‐seeking tendencies relate to HIE. Administration of psychometric inventories is one way to quantify these traits and allow athletes to self‐report the degree to which they possess them. Several validated inventories exist that measure either aggression or sensation seeking, including the Competitive Anger and Aggressiveness Scale (CAAS) and the Brief Sensation Seeking Scale (BSSS) (Hoyle et al., 2002; Maxwell & Moores, 2007). The CAAS is a survey specifically designed and validated to assess sports‐related aggression. The CAAS has previously been used to define aggression in youth ice hockey players, in a study that found players with higher CAAS scores sustained higher magnitudes of mean rotational acceleration per impact (Schmidt et al., 2016). The BSSS is a short survey validated to assess sensation‐seeking tendencies. Although it is not sport specific like the CAAS, higher BSSS scores were found to increase the risk of sports‐related concussion in a large cohort of collegiate athletes. Both inventories thus show promise in quantifying athletes’ baseline behavioral tendencies that may relate to HIE sustained individually.

Therefore, the primary purpose of this study was to examine the relationships between HIE and outcomes from a novel behavioral assessment battery of aggression (CAAS and PM) and sensation‐seeking (BSSS) tendencies in a single season of collegiate ice hockey. We performed a secondary, exploratory analysis to examine the relationships between these behaviors and MRI‐based measures of neural structure, as the objective nature of neuroimaging can add valuable information on the underlying neural mechanisms that might explain aggression and sensation seeking. Both aggression and sensation seeking are linked to activation in structures of the limbic system and frontal cortex, particularly the amygdala, orbitofrontal cortex (OFC), anterior cingulate cortex (ACC), and ventromedial prefrontal cortex (vmPFC) (Cupaioli et al., 2021; Joseph et al., 2009). Structural neuroimaging analyses have identified that repetitive head impacts may affect structural connectivity and volume of the amygdala, but there is less evidence describing the other structures (Lepage et al., 2019; McAllister et al., 2014). We hypothesized that (1) there would be a positive relationship between the number of head impacts sustained in a season and our measures of aggression and sensation seeking, and (2) there would be a positive relationship between our MRI‐based measures of brain structure and our measures of aggression and sensation seeking.

## 2 METHODS

Eighteen Division I male collegiate club ice hockey players participated in this study that coincided with the 2017–2018 or 2018–2019 seasons. Participants were included in the study if they were healthy and able to fully participate in the hockey season. Goaltenders were excluded from this study as they do not regularly sustain head impacts. For players who participated in this study over both seasons, only their most recent season of data was used. All players completed the CAAS and BSSS during their baseline concussion testing session at the start of the season. For our secondary MRI‐based analysis, only those participants who completed the baseline neuroimaging protocol were included (*n* = 15). Three participants were excluded from imaging analyses due to shrapnel in the eye or claustrophobia. This study was approved by the University of Delaware Institutional Review Board, in accordance with the requirements set by the Ethical Principles and Guidelines for the Protection of Human Subjects of Research (“Belmont Report”) and the Code of Federal Regulations. All participants provided written informed consent.

This study utilized three outcome measures to assess aggression and sensation seeking: PM normalized to the number of games played (PM_norm_), the CAAS, and the BSSS. PM_norm_ were obtained from game statistics of total PM and games played were taken from the official game records uploaded to the American Collegiate Hockey Association archives (ACHA, n.d.) and defined as the number of cumulative PM sustained in a season divided by the number of games played $\left(PM_{\mathrm{norm}}=\;\frac{\mathrm{PM}}{\text{Games}\;\text{played}}\right)$. The CAAS is a 12‐item questionnaire that measures athletes’ perceived aggressiveness and anger during athletic competition (Maxwell & Moores, 2007). Participants self‐reported their answers on a 7‐point Likert scale, ranging from 1 (*strongly disagree*) to 7 (*strongly agree*), for a total score between 12 and 72, in which higher scores denoted more self‐reported anger and aggression. The BSSS is a valid and reliable eight‐question measure of trait sensation‐seeking tendencies, adapted from the Sensation‐Seeking Scale—Form V (Hoyle et al., 2002). Questions were scored on a 5‐point scale (“*strongly disagree*” to “*strongly agree*”), where higher scores indicated greater sensation‐seeking tendencies.

To quantify HIE, participants wore Smart Impact Monitors (SIM, Triax, Norwalk, CT) at all practice and home games in a season (Cummiskey et al., 2017). Head impacts >10 g were recorded and wirelessly transmitted to a computer with Triax software installed and overseen by a member of the research staff. Each session of each season was video recorded and the film was used to confirm true head impacts and remove false impacts (Patton et al., 2020). The cumulative number of all true head impacts registered was compiled per player each season for our measure of HIE.

All MRI data were acquired at the University of Delaware Center for Biomedical and Brain Imaging using a Siemens Prisma 3T scanner (Siemens Healthineers, Erlangen, Germany) for our secondary analyses. For volumetric analysis and anatomical localization, a T1‐weighted three‐dimensional magnetization prepared rapid acquisition gradient recalled echo (MPRAGE) anatomical scan (repetition time (TR)/echo time (TE)/inversion time (TI) = 2530/3.36/900 ms; 1.0 mm isotropic resolution) was collected. Magnetic resonance elastography (MRE) data were collected with a three‐dimensional multislab, multishot spiral sequence (Johnson, Holtrop, et al., 2016) with the following parameters: TR/TE = 2133/70 ms; 240 × 240 mm^2^ field of view; 120 × 120 matrix; 64 slices; and 2.0 mm isotropic resolution. Vibrations at 60 Hz were generated by the Resoundant pneumatic actuator system and delivered to the head via soft pillow driver (Resoundant, Inc., Rochester, MN). A nonlinear inversion algorithm (NLI) was used to estimate our outcome measures of viscoelastic shear stiffness and damping ratio from measured MRE displacement fields. NLI estimates the complex shear modulus, $G\;=G^{\prime }\;+iG^{\prime \prime }$, where $G^{\prime }\;$ represents the storage modulus and $G^{\prime \prime }$ represents the loss modulus of the brain tissue (McGarry et al., 2012; Van Houten et al., 2001). Both were used to calculate viscoelastic shear stiffness $\left(\mu =\frac{2{\left|G\right|}^{2}}{G^{\prime }+\left|G\right|}\right)$ and damping ratio $\left(\xi =\frac{G^{\prime \prime }}{2G^{\prime }}\right)$ (Manduca et al., 2001; McGarry et al., 2012; Van Houten et al., 2001). Stiffness and damping ratio reflect the composition and organization of brain microstructure, and both have been shown to be sensitive to brain health (Hiscox et al., 2021; McIlvain et al., 2020; Schwarb et al., 2016).

For both volumetric and MRE analyses, we chose a priori to evaluate the bilateral amygdala, thalamus, hippocampus, OFC, vmPFC, and ACC as our regions of interest (ROIs), as these are structures that have been consistently reported as neural structures that contribute to both sensation seeking and aggression. Volume of ROIs was extracted from T1‐weighted images using Freesurfer following a visual inspection of fit quality and completion of the automatic segmentation (Buckner et al., 2004; Fischl, 2012; Fischl & Dale, 1999). Images of the Freesurfer segmentations for each subject are included as Figure S1. OFC measures were obtained by combining the values of the medial and lateral OFC. vmPFC measures were obtained by combining the values of the rostral middle frontal cortex and rostral anterior cingulate cortex. ACC measures were obtained by combining the values of the caudal and rostral anterior cingulate cortex. Per Freesurfer recommendations, volume measures were normalized to intracranial volumes (Buckner et al., 2004). Additionally, we incorporated a priori spatial information during MRE mechanical property estimation using NLI with soft prior regularization (SPR), which has been used to reduce uncertainty in measures (Johnson, Schwarb, et al., 2016; McGarry et al., 2013). Masks of our ROIs were provided separately to NLI for use in SPR by registering MPRAGE images to their corresponding MRE magnitude images using the FLIRT tool within FMRIB Software Library (FSL) (Jenkinson et al., 2012).

For our primary analysis of how our behavioral assessment battery relates to HIE, Pearson correlations were performed between number of head impacts sustained, total CAAS score, BSSS score, and PM_norm_. For our secondary, exploratory analysis of how structural and mechanical brain properties are related to behavior and HIE, a separate Pearson correlation was also performed between number of head impacts, CAAS scores, BSSS scores, PM_norm_, and volume, stiffness, and damping ratio for each of our ROIs (amygdala, thalamus, OFC, ACC, vmPFC). Significant relationships were determined at *p *< .05 for all analyses. Bonferroni correction was applied to correct for multiple comparisons and corrected *p*‐values are also reported. All statistical analyses were performed using IBM SPSS Statistics for Mac, version 26.0.0 (IBM Corp., Armonk, NY).

## 3 RESULTS

**FIGURE 1:**
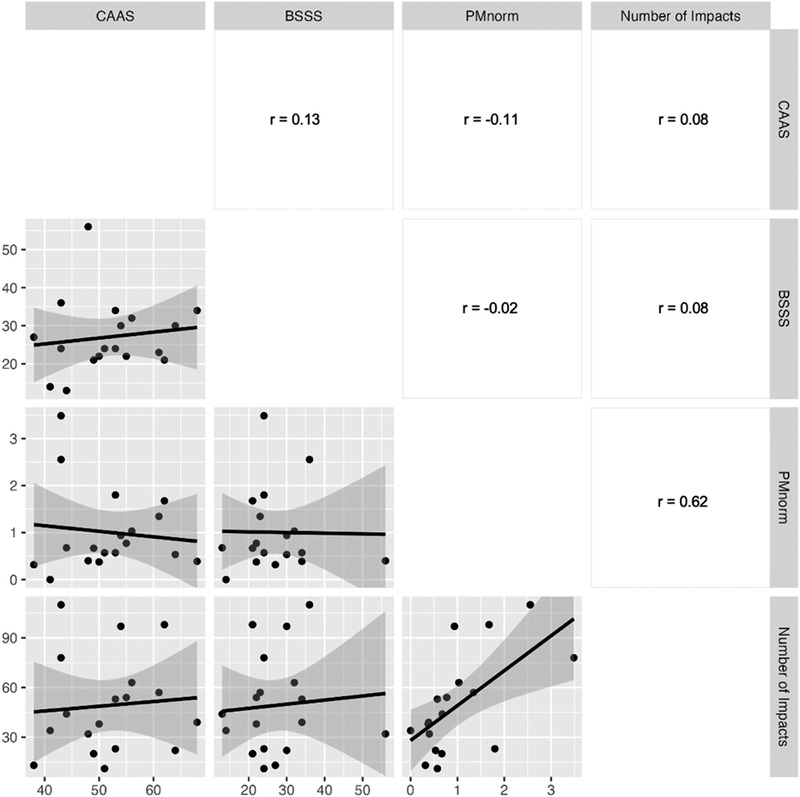
Correlation matrix between head impact exposure, aggression, and sensation seeking

Descriptive statistics for CAAS, BSSS, PM_norm_, and number of head impacts are reported in Table 1 for all participants (*n* = 18). PM_norm_ represented the number of PM sustained per number of games played, where the mean number of PM was 22.1 ± 20.8 min (range: 0—69 min) and the mean number of games played in our cohort was 23.8 ± 10.3 (range: 0–38). A significant relationship was found between (*r* = .65, *p *< .01, *p‐*corrected < .01) PM_norm_ and number of head impacts sustained in our cohort, such that more PM_norm_ were related to more head impacts sustained (Figure 1). Only nonsignificant relationships between CAAS, BSSS, and number of impacts were found (all *p* > .05; Figure 1).

**TABLE 1:** Mean values ± standard deviations of our behavior‐ and neuroimaging‐based measures

Measure	Variable	Value	ROI
Behavioral	CAAS	51.6 ± 8.5	
	BSSS	26.8 ± 9.9	
	PM_norm_	1.0 ± 0.9	
	Number of impacts	48.4 ± 30.7	
Neuroimaging	Stiffness (kPa)	3.33 ± 0.33	Amygdala
		2.90 ± 0.40	Hippocampus
		3.85 ± 0.32	Thalamus
		4.36 ± 0.37	ACC
		3.21 ± 0.30	OFC
		3.21 ± 0.18	vmPFC
	Damping ratio	0.250 ± 0.03	Amygdala
		0.249 ± 0.03	Hippocampus
		0.257 ± 0.04	Thalamus
		0.188 ± 0.02	ACC
		0.292 ± 0.04	OFC
		0.302 ± 0.03	vmPFC
	Volume (cm^3^)	1.85 ± 0.01	Amygdala
		4.41 ± 0.02	Hippocampus
		7.97 ± 0.50	Thalamus
		4.89 ± 0.65	ACC
		14.65 ± 1.04	OFC
		21.22 ± 2.04	vmPFC

*Note*: CAAS, BSSS, and PM_norm_ represent our behavioral battery. Number of impacts is a measure of head impact exposure. Neuroimaging data were measured in a priori selected ROIs. Stiffness and damping ratio are MRE‐based measures; volume is a volumetric MRI‐based measure.

We used preseason values of volume, stiffness, and damping ratio of our ROIs to explore the relationships between neural structure and CAAS, BSSS, PM_norm_, and number of head impacts (Table 2). Descriptive statistics for our neuroimaging measures are presented in Table 1. All participants’ volumetric data are represented, but MRE data from five of the 15 participants were excluded due to poor signal‐to‐noise ratio (McGarry et al., 2011). We found significant relationships between brain mechanical properties—both stiffness and damping ratio—in several of our ROIs and the CAAS, BSSS, and number of impacts (Figures S2 and S3). Though these relationships were not still statistically significant at *p *< .05 after correction for multiple comparisons, these are the first data to describe how brain stiffness may relate to behavioral traits in athletes. BSSS scores were related to stiffness of both the amygdala (*r* = .69, *p *= .04, *p‐*corrected = .22) and thalamus (*r* = .72, *p *= .02, *p‐*corrected = .11), and inversely related to vmPFC damping ratio (*r* = −.64, *p *= .05, *p‐*corrected = .26). CAAS scores were also inversely related to hippocampal damping ratio (*r* = −.65, *p *= .04, *p‐*corrected = .22). A relationship was found between number of impacts and thalamic damping ratio (*r* = .75, *p *= .03, *p‐*corrected = .17), such that a player with higher damping ratio experienced more head impacts. No relationships were found between volumetric measures in our ROIs and any behavioral variables.

**TABLE 2:** Correlation matrix between imaging and behavioral measures with Pearson correlation coefficients, *r*, for each relationship

	ROI	Impacts	CAAS	BSSS	PM_norm_
Stiffness	ACC	−0.07	0.17	0.48	−0.15
	Amygdala	0.11	0.10	0.69	0.34
	Hippocampus	0.02	0.11	0.19	0.35
	OFC	−0.10	0.36	0.13	−0.22
	Thalamus	0.15	0.53	0.72	−0.26
	vmPFC	−0.05	−0.06	0.17	−0.11
Damping ratio	ACC	−0.07	0.36	0.64	−0.14
	Amygdala	0.01	−0.37	0.28	−0.34
	Hippocampus	0.10	−0.68	−0.07	−0.17
	OFC	−0.08	−0.45	−0.18	−0.04
	Thalamus	0.75	0.31	0.15	0.28
	vmPFC	0.14	0.29	−0.65	−0.24
Volume	ACC	0.51	0.23	−0.35	0.05
	Amygdala	−0.04	−0.46	−0.37	0.14
	Hippocampus	−0.24	0.10	−0.15	−0.19
	OFC	0.31	0.12	−0.44	0.10
	Thalamus	0.33	0.04	0.27	−0.22

## 4 DISCUSSION

This study utilized a novel battery of behavioral inventories to assess the relationships among aggression, sensation‐seeking tendencies, brain structure, and HIE over a season in men's collegiate club ice hockey players. The main finding of this study was a strong, statistically significant correlation (*r* = .62) between head impact frequency and PM sustained, suggesting a relationship between more aggressive playing style and sustaining a greater number of head impacts over a season. Our secondary, exploratory analysis revealed that viscoelastic mechanical properties of several neural structures known to regulate aggression and sensation seeking, especially the thalamus, were related to components of our behavioral battery. We did not observe any significant relationships between volume of these structures and our battery, suggesting that measuring the mechanical properties may be a more sensitive measure to use in further understanding the link between personality traits, behavior, and brain structure.

This was the first study to describe relationships between a behavioral assessment battery including a measure of aggression directly related to playing style (PM_norm_), a sport‐specific inventory of perceived aggression in playing style (CAAS), and a broad inventory of perceived sensation‐seeking tendencies (BSSS) and HIE. We found that only our most direct measure of aggression in ice hockey, cumulative PM_norm_ accrued over the season, was related to HIE. It is worth noting that our player with the greatest number of PM did not play in the most games, which supports our decision to include PM_norm_ as a measure over just PM or games played individually. While this study cannot define any causal relationship between aggression through PM and HIE, previous studies have individually identified the majority of PM as “aggressive” (e.g., boarding), compared to those not considered aggressive (e.g., offsides), as well as that most PM result from body checking (Bushman & Wells, 1998; Carré & McCormick, 2008; Emery et al., 2011; Gee & Leith, 2007). Given that checking is the primary mechanism for sustaining RHI in hockey, this evidence supports the relationship found between aggression and HIE in this study.

We did not find any significant relationship between a player's perceived competitive aggressive tendencies (CAAS) and HIE, suggesting that using the CAAS may not be useful for understanding behaviors that result in HIE. However, there are several alternate explanations to consider as to why we did not find a relationship between CAAS scores and HIE. In general, there is a high base rate of aggression found in hockey players and therefore there may be a ceiling effect to the CAAS (Bushman & Wells, 1998). It is possible that our participants perceived themselves as similarly aggressive, and thus not enough variability was reported through CAAS scores to have elucidated a relationship to HIE. The frustration‐aggression hypothesis may also help explain why we did not find a significant relationship between HIE and CAAS, considering that we did find a significant relationship between HIE and PM_norm_. It states that frustration will occur when an individual cannot achieve a particular goal, and increase the chances he or she will vent frustration in an aggressive manner that is usually directed toward the source of frustration (Berkowitz, 1989). In hockey terms, this means a player would need to be placed in a situation that causes frustration in order to display aggression, which would not be captured through a measure of perceived aggression, like the CAAS, though it could be captured with a direct measure from playing, such as PM_norm_ (Gee & Leith, 2007).

The effects of sensation seeking have not been as frequently studied as aggression in hockey players, but we chose to include this measure because higher reported sensation seeking may be a risk factor of sports‐related concussion in collegiate athletes (Liebel et al., 2020; Osborn et al., 2009). We did not find any significant relationship between BSSS and HIE in our cohort, suggesting, like the CAAS, that the BSSS may not be a useful tool in this application. In this case, the BSSS was not specific to sports competition and instead quantified the perceived desire to seek out certain experiences, but not engagement in directed acts. In a hockey context, this may or may not include engaging in body contact and therefore support why we did not find a relationship between BSSS scores and HIE. However, it may still be worth monitoring BSSS scores in hockey athletes despite this lack of relationship, as higher BSSS scores have been reported as related to increased risk of sustaining a concussion (Liebel et al., 2020).

In addition to assessing these relationships, we also chose to explore how differences in MRI‐measured brain structure may affect both scores on our behavioral battery and HIE in a season of ice hockey. The objective nature of neuroimaging, as compared to self‐reported inventories or behavioral assessments, can aid in solidifying our understanding of the mechanisms linking aggression or sensation seeking and HIE. Furthermore, it can help us to understand who may be more likely to display such traits, and therefore at risk of the consequences associated with those traits. We found that baseline values of both stiffness and damping ratio were related to both aggression and sensation seeking, but structural volume was not. Though these relationships did not exhibit significant corrected *p*‐values after correction of multiple comparisons, the results from this exploratory analysis are consistent with previous brain MRE literature, which has reported that changes in mechanical properties of neural tissue can occur in the absence of changes in volume or thickness (Hiscox et al., 2021).

Little is known about the relationship between brain stiffness and personality traits, but adolescents who engaged in more risky behaviors trended toward having greater stiffness in neural structures related to risk taking (McIlvain et al., 2020). We found thalamic stiffness was related to sensation seeking, such that greater stiffness was related to higher BSSS scores (*r* = .71). In general, higher stiffness is thought to reflect higher tissue integrity and neural health, as it results from a denser glial matrix or healthier myelin sheaths (Johnson & Telzer, 2018). Our results suggest that a thalamus with greater integrity may be more likely to exhibit a greater degree of sensation‐seeking traits. We also found a significant relationship between amygdala stiffness and BSSS scores (*r* = .65), which may be explained by similar reasoning. The amygdala communicates with the cortex in order to make decisions regarding emotion, which could explain why both greater stiffness of the amygdala and thalamus related to more pronounced perceptions of sensation seeking in our participants (Baas et al., 2004; Bell & Shine, 2016; Sherman, 2007).

In addition to stiffness, we also found significant relationships between BSSS scores and damping ratio. While stiffness is thought to reflect microstructural composition, MRE literature suggests that damping ratio reflects microstructural organization and is more correlated with brain function (Johnson et al., 2018; Sack et al., 2013; Schwarb et al., 2016). BSSS scores were inversely related to vmPFC damping ratio, such that higher BSSS was related to lower vmPFC damping ratio. The vmPFC has a more targeted role in controlling an individual's response to emotion, whereas the amygdala evaluates emotions from stimuli, and the thalamus communicates with the two (Baas et al., 2004; Bell & Shine, 2016; Sherman, 2007). It is fitting that we would find a relationship with damping ratio in the more functionally specific vmPFC and BSSS, as it has been suggested that damping ratio is generally more correlated with brain function than stiffness due to heightened sensitivity to individual differences (Hiscox et al., 2021).

Altogether, our results provide initial support toward the clinical utility of monitoring PM to determine which hockey players may be at risk for greater HIE due to heightened aggressive behaviors. Finding interventions that lower aggression, such as behavioral modification through psychoeducation, may be a novel approach to lower HIE and preventing subsequent neuropathologies (Bazarian et al., 2014; McKee et al., 2009; Montenigro et al., 2017). No studies have yet examined how using psychoeducation to modify traits such as aggression affects HIE or head injury risk in collegiate athletes, but behavioral modification programs targeting football‐specific physical behavior (e.g., tackling) have been successful in reducing HIE in youth athletes (Champagne et al., 2019; Combs et al., 2019). Interestingly, despite belief that aggression will benefit individual or team performance, this relationship has not been proven (Gee & Leith, 2007). This is an exciting finding, as lowering aggression may help keep athletes safer without impairment or negatively affecting performance.

Despite our novel findings, there are several limitations to this study that may affect generalizability of our results. First, we were limited to a small sample based on the size of the ice hockey team studied, but our sample size was comparable to existing studies that have also utilized our more advanced measures of MRI and head impact monitoring. We were limited in our ability to collect head impact data from games away from the home stadium, although it is likely that individual's HIE would trend in a similar direction, thus we expect our measure of HIE to accurately reflect the total exposure also incorporating away games. Additionally, our results may have been affected by the inherent limitations associated with self‐reported measures. Due to the subjective nature of such measures, it is not possible to know whether participants responded honestly or accurately to the questions on the CAAS or BSSS, though we were able to include an objective measure of aggression (PM_norm_) to partially combat this limitation. Although we ran our secondary analysis as exploratory, we were left with a low MRE sample due to insufficiencies in data quality and thus unequal samples between the two analyses. However, this is the first study to describe brain viscoelasticity in relation to either athletes or repetitive head impacts and may serve as preliminary evidence for further use of MRE in assessing the causes and effects of HIE. As brain MRE is a relatively new neuroimaging modality, future studies should also consider how the relationships between MRE and behavior compare to those between behavior and other imaging methods sensitive to tissue microstructure (e.g., diffusion tensor imaging) to develop a comprehensive understanding of how brain structure may influence expression of personality traits.

## 5 CONCLUSION

This study provides important results that help our understanding of HIE risk, as it is the first study to examine how personality traits, brain structure, and HIE are related. Our results suggest that monitoring PM may be useful in determining athletes at risk for greater HIE, and that behavior modification targeting aggression may be useful in reducing HIE. However, our design and methods should be considered as preliminary evidence for future research with larger cohorts on this topic.

## CONFLICT OF INTEREST

The authors declare no conflict of interest.

### PEER REVIEW

The peer review history for this article is available at https://publons.com/publon/10.1002/brb3.2627.

## Supporting information

Figure S1. Freesurfer segmentation of the amygdala for all participants.
Figure S2: Scatterplots of significant ROI stiffness (kPa) and behavioral relationships.
Figure S3. Scatterplots of significant ROI damping ratio and behavioral relationships.

## Data Availability

Data available on request from the authors.

## References

[brb32627-bib-0001] Agel, J. , Dompier, T. P. , Dick, R. , & Marshall, S. W. (2007). Descriptive epidemiology of collegiate men's ice hockey injuries: National Collegiate Athletic Association injury surveillance system, 1988–1989 through 2003–2004. Journal of Athletic Training, 42(2), 241–248. 17710172 PMC1941284

[brb32627-bib-0002] American Collegiate Hockey Association (ACHA) . (n.d.). Team overview, M1 University of Delaware . http://achahockey.org/view/achahockey/men‐s‐d1/teams‐361?type=overview&level=team&id=508494&division_id=108748&league_id=1800&conference_id=1150

[brb32627-bib-0003] Baas, D. , Aleman, A. , & Kahn, R. S. (2004). Lateralization of amygdala activation: A systematic review of functional neuroimaging studies. Brain Research Reviews, 45(2), 96–103. 10.1016/j.brainresrev.2004.02.004 15145620

[brb32627-bib-0004] Bazarian, J. J. , Zhu, T. , Zhong, J. , Janigro, D. , Rozen, E. , Roberts, A. , Javien, H. , Merchant‐Borna, K. , Abar, B. , & Blackman, E. G. (2014). Persistent, long‐term cerebral white matter changes after sports‐related repetitive head impacts. PLoS ONE, 9(4), e94734. 10.1371/journal.pone.0094734 24740265 PMC3989251

[brb32627-bib-0005] Beckwith, J. G. , Greenwald, R. M. , Chu, J. J. , Crisco, J. J. , Rowson, S. , Duma, S. M. , Broglio, S. P. , McAllister, T. W. , Guskiewicz, K. M. , Mihalik, J. P. , Anderson, S. , Schnebel, B. , Brolinson, P. G. , & Collins, M. W. (2013). Timing of concussion diagnosis is related to head impact exposure prior to injury. Medicine and Science in Sports and Exercise, 45(4), 747–754. 10.1249/MSS.0b013e3182793067 23135364 PMC3605273

[brb32627-bib-0006] Bell, P. T. , & Shine, J. M. (2016). Subcortical contributions to large‐scale network communication. Neuroscience & Biobehavioral Reviews, 71, 313–322. 10.1016/j.neubiorev.2016.08.036 27590830

[brb32627-bib-0007] Berkowitz, L. (1989). Frustration‐aggression hypothesis: Examination and reformulation. Psychological Bulletin, 106(1), 59–73. 10.1037/0033-2909.106.1.59 2667009

[brb32627-bib-0008] Buckner, R. L. , Head, D. , Parker, J. , Fotenos, A. F. , Marcus, D. , Morris, J. C. , & Snyder, A. Z. (2004). A unified approach for morphometric and functional data analysis in young, old, and demented adults using automated atlas‐based head size normalization: Reliability and validation against manual measurement of total intracranial volume. Neuroimage, 23(2), 724–738. 10.1016/j.neuroimage.2004.06.018 15488422

[brb32627-bib-0009] Bushman, B. J. , & Wells, G. L. (1998). Trait aggressiveness and hockey penalties: Predicting hot tempers on the ice. Journal of Applied Psychology, 83(6), 969–974. 10.1037/0021-9010.83.6.969 9885201

[brb32627-bib-0010] Carré, J. M. , & McCormick, C. M. (2008). In your face: Facial metrics predict aggressive behaviour in the laboratory and in varsity and professional hockey players. Proceedings of the Royal Society B: Biological Sciences, 275(1651), 2651–2656. 10.1098/rspb.2008.0873 PMC2570531 18713717

[brb32627-bib-0011] Champagne, A. A. , Distefano, V. , Boulanger, M. M. , Magee, B. , Coverdale, N. S. , Gallucci, D. , Guskiewicz, K. , & Cook, D. J. (2019). Data‐informed intervention improves football technique and reduces head impacts. Medicine and Science in Sports and Exercise, 51(11), 2366–2374. 10.1249/MSS.0000000000002046 31269008 PMC7028524

[brb32627-bib-0012] Combs, P. R. , Ford, C. B. , Nocera, M. , Campbell, K. R. , Marshall, S. W. , Mihalik, J. , Register‐Mihalik, J. , & Guskiewicz, K. M. (2019). Biomechanic data‐driven behavior modification to reduce concussion risk in high school football athletes. Orthopaedic Journal of Sports Medicine, 7(3), 2325967119S0010. 10.1177/2325967119s00105

[brb32627-bib-0013] Cummiskey, B. , Schiffmiller, D. , Talavage, T. M. , Meyer, J. J. , Adams, D. , & Nauman, E. A. (2017). Reliability and accuracy of helmet‐mounted and head‐mounted devices used to measure head accelerations. Proceedings of the Institution of Mechanical Engineers, Part P: Journal of Sports Engineering and Technology, 231(2), 144–153. 10.1177/1754337116658395

[brb32627-bib-0014] Cupaioli, F. A. , Zucca, F. A. , Caporale, C. , Lesch, K. P. , Passamonti, L. , & Zecca, L. (2021). The neurobiology of human aggressive behavior: Neuroimaging, genetic, and neurochemical aspects. Progress in Neuro‐Psychopharmacology and Biological Psychiatry, 106, 110059. 10.1016/j.pnpbp.2020.110059 32822763

[brb32627-bib-0015] Cusimano, M. D. , Nastis, S. , & Bhsc, L. Z. (2013). Effectiveness of interventions to reduce aggression and injuries among ice hockey players: A systematic review. Canadian Medical Association Journal, 185(1), 57–69. 10.1503/cmaj PMC3537813 23209118

[brb32627-bib-0016] Emery, C. A. , Kang, J. , Schneider, K. J. , & Meeuwisse, W. H. (2011). Risk of injury and concussion associated with team performance and penalty minutes in competitive youth ice hockey. British Journal of Sports Medicine, 45(16), 1289–1293. 10.1136/bjsports-2011-090538 22117019

[brb32627-bib-0017] Emery, C. A. , & Meeuwisse, W. H. (2006). Injury rates, risk factors, and mechanisms of injury in minor hockey. The American Journal of Sports Medicine, 34(12), 1960–1969. 10.1177/0363546506290061 16861577

[brb32627-bib-0018] Emery, C. , Palacios‐Derflingher, L. , Black, A. M. , Eliason, P. , Krolikowski, M. , Spencer, N. , Kozak, S. , Schneider, K. J. , Babul, S. , Mrazik, M. , Lebrun, C. M. , Goulet, C. , Macpherson, A. , & Hagel, B. E. (2019). Does disallowing body checking in non‐elite 13‐ to 14‐year‐old ice hockey leagues reduce rates of injury and concussion? A cohort study in two Canadian provinces. British Journal of Sports Medicine, 54, 414–420. 10.1136/bjsports-2019-101092 31492676

[brb32627-bib-0019] Fischl, B. , & Dale, A. M. (1999). Measuring the thickness of the human cerebral cortex. Proceedings of the National Academy of Sciences of the United States of America, 97(20), 11050–11055. 10.1073/pnas.200033797 PMC27146 10984517

[brb32627-bib-0020] Fischl, B. (2012). FreeSurfer. Neuroimage, 62(2), 774–781. 10.1016/j.neuroimage.2012.01.021 22248573 PMC3685476

[brb32627-bib-0021] Gee, C. J. , & Leith, L. M. (2007). Aggressive behavior in professional ice hockey: A cross‐cultural comparison of North American and European born NHL players. Psychology of Sport and Exercise, 8(4), 567–583. 10.1016/j.psychsport.2006.06.006

[brb32627-bib-0022] Hiscox, L. V. , Schwarb, H. , McGarry, M. D. J. , & Johnson, C. L. (2021). Aging brain mechanics: Progress and promise of magnetic resonance elastography. Neuroimage, 232, 117889. 10.1016/j.neuroimage.2021.117889 33617995 PMC8251510

[brb32627-bib-0023] Hoyle, R. H. , Stephenson, M. T. , Palmgreen, P. , Pugzles, E. , & Donohew, R. L. (2002). Reliability and validity of a brief measure of sensation seeking. Personality and Individual Differences, 32(3), 401–414.

[brb32627-bib-0024] Jenkinson, M. , Beckmann, C. F. , Behrens, T. E. J. , Woolrich, M. W. , & Smith, S. M. (2012). FSL. Neuroimage, 62(2), 782–790. 10.1016/j.neuroimage.2011.09.015 21979382

[brb32627-bib-0025] Johnson, C. L. , Holtrop, J. L. , Anderson, A. T. , & Sutton, B. P. (2016). Brain MR elastography with multiband excitation and nonlinear motion‐induced phase error correction . Proceedings of the 24th Annual Meeting of the International Society for Magnetic Resonance in Medicine, 1951.

[brb32627-bib-0026] Johnson, C. L. , Schwarb, H. , McGarry, D. J. , Anderson, A. T. , Huesmann, G. R. , Sutton, B. P. , & Cohen, N. J. (2016). Viscoelasticity of subcortical gray matter structures. Human Brain Mapping, 37(12), 4221–4233. 10.1002/hbm.23314 27401228 PMC5118063

[brb32627-bib-0027] Johnson, C. L. , Schwarb, H. , Horecka, K. M. , McGarry, M. D. J. , Hillman, C. H. , Kramer, A. F. , Cohen, N. J. , & Barbey, A. K. (2018). Double dissociation of structure‐function relationships in memory and fluid intelligence observed with magnetic resonance elastography. Neuroimage, 171, 99–106. 10.1016/j.neuroimage.2018.01.007.Double 29317306 PMC5857428

[brb32627-bib-0028] Johnson, C. L. , & Telzer, E. H. (2018). Magnetic resonance elastography for examining developmental changes in the mechanical properties of the brain. Developmental Cognitive Neuroscience, 33, 176–181. 10.1016/j.dcn.2017.08.010 29239832 PMC5832528

[brb32627-bib-0029] Joseph, J. E. , Liu, X. , Jiang, Y. , Lynam, D. , & Kelly, T. H. (2009). Neural correlates of emotional reactivity in sensation seeking. Psychological Science, 20(2), 215–223. 10.1111/j.1467-9280.2009.02283.x 19222814 PMC3150539

[brb32627-bib-0030] Lepage, C. , Muehlmann, M. , Tripodis, Y. , Hufschmidt, J. , Stamm, J. , Green, K. , Wrobel, P. , Schultz, V. , Weir, I. , Alosco, M. L. , Baugh, C. M. , Fritts, N. G. , Martin, B. M. , Chaisson, C. , Coleman, M. J. , Lin, A. P. , Pasternak, O. , Makris, N. , Stern, R. A. , … Koerte, I. K. (2019). Limbic system structure volumes and associated neurocognitive functioning in former NFL players. Brain Imaging and Behavior, 13(3), 725–734. 10.1007/s11682-018-9895-z 29779184 PMC6854905

[brb32627-bib-0031] Liebel, S. W. , Van Pelt, K. L. , Garcia, G. G. P. , Czerniak, L. L. , McCrea, M. A. , McAllister, T. W. , & Broglio, S. P. (2020). The relationship between sport‐related concussion and sensation‐seeking. International Journal of Molecular Sciences, 21(23), 1–12. 10.3390/ijms21239097 PMC7729784 33265913

[brb32627-bib-0032] Mainwaring, L. , Pennock, K. M. F. , Mylabathula, S. , & Alavie, B. Z. (2018). Subconcussive head impacts in sport : A systematic review of the evidence. International Journal of Psychophysiology, 132, 39–54. 10.1016/j.ijpsycho.2018.01.007 29402530

[brb32627-bib-0033] Manduca, A. , Oliphant, T. E. , Dresner, M. A. , Mahowald, J. L. , Kruse, S. A. , Amromin, E. , Felmlee, J. P. , Greenleaf, J. F. , & Ehman, R. L. (2001). Magnetic resonance elastography: Non‐invasive mapping of tissue elasticity. Medical Image Analysis, 5(4), 237–254. 10.1016/S1361-8415(00)00039-6 11731304

[brb32627-bib-0034] Maxwell, J. P. , & Moores, E. (2007). The development of a short scale measuring aggressiveness and anger in competitive athletes. Psychology of Sport and Exercise, 8, 179–193. 10.1016/j.psychsport.2006.03.002

[brb32627-bib-0035] McAllister, T. W. , Ford, J. C. , Flashman, L. a. , Maerlender, A. , Greenwald, R. M. , Beckwith, J. G. , Bolander, R. P. , Tosteson, T. D. , Turco, J. H. , Raman, R. , & Jain, S. (2014). Effect of head impacts on diffusivity measures in a cohort of collegiate contact sport athletes. Neurology, 82(1), 63–69. 10.1212/01.wnl.0000438220.16190.42 24336143 PMC3873621

[brb32627-bib-0036] McGarry, M. D. J. , Van Houten, E. E. W. , Johnson, C. L. , Georgiadis, J. G. , Sutton, B. P. , Weaver, J. B. , & Paulsen, K. D. (2012). Multiresolution MR elastography using nonlinear inversion. Medical Physics, 39(10), 6388–6396. 10.1118/1.4754649 23039674 PMC3477197

[brb32627-bib-0037] McGarry, M. D. J. , Van Houten, E. E. W. , Perrĩez, P. R. , Pattison, A. J. , Weaver, J. B. , & Paulsen, K. D. (2011). An octahedral shear strain‐based measure of SNR for 3D MR elastography. Physics in Medicine and Biology, 56(13), N153–N164. 10.1088/0031-9155/56/13/N02 21654044 PMC3172714

[brb32627-bib-0038] McGarry, M. , Johnson, C. L. , Sutton, B. P. , Van Houten, E. E. W. , Georgiadis, J. G. , Weaver, J. B. , & Paulsen, K. D. (2013). Including spatial information in nonlinear inversion MR elastography using soft prior regularization. IEEE Transactions on Medical Imaging, 32(10), 1901–1909. 10.1109/TMI.2013.2268978 23797239 PMC4107367

[brb32627-bib-0039] McIlvain, G. , Clements, R. G. , Magoon, E. M. , Spielberg, J. M. , Telzer, H. , & Johnson, C. L. (2020). Viscoelasticity of reward and control systems in adolescent risk taking. Neuroimage, 215, 116850. 10.1016/j.neuroimage.2020.116850 32298793 PMC7292790

[brb32627-bib-0040] McKee, A. C. , Cantu, R. C. , Nowinski, C. J. , Hedley‐Whyte, E. T. , Gavett, B. E. , Budson, A. E. , Santini, V. E. , Lee, H.‐S. , Kubilus, C. A. , & Stern, R. A. (2009). Chronic traumatic encephalopathy in athletes: Progressive tauopathy after repetitive head injury. Journal of Neuropathology and Experimental Neurology, 68(7), 709–735. 10.1097/NEN.0b013e3181a9d503 19535999 PMC2945234

[brb32627-bib-0041] Montenigro, P. H. , Alosco, M. L. , Martin, B. M. , Daneshvar, D. H. , Mez, J. , Chaisson, C. E. , Nowinski, C. J. , Au, R. , McKee, A. C. , Cantu, R. C. , McClean, M. D. , Stern, R. A. , & Tripodis, Y. (2017). Cumulative head impact exposure predicts later‐life depression, apathy, executive dysfunction, and cognitive impairment in former high school and college football players. Journal of Neurotrauma, 34(7), 1490–1490. 10.1089/neu.2016.4539 27029716 PMC5220530

[brb32627-bib-0042] Osborn, Z. H. , Blanton, P. D. , & Schwebel, D. C. (2009). Personality and injury risk among professional hockey players. Journal of Injury & Violence Research, 1(1), 15–19. 10.5249/jivr.v1i1.8 21483186 PMC3134906

[brb32627-bib-0043] Patton, D. A. , Huber, C. M. , Mcdonald, C. C. , Margulies, S. S. , Master, C. L. , & Arbogast, K. B. (2020). Video confirmation of head impact sensor data from high school soccer players. The American Journal of Sports Medicine, 48(5), 1246–1253. 10.1177/0363546520906406 32130020 PMC7405551

[brb32627-bib-0044] Pedersen, D. (2007). Perceived aggression in sports and its relation to willingness to participate and perceived risk of injury. Perceptual and Motor Skills, 104, 201–211. 10.2466/pms.104.1.201-211 17450982

[brb32627-bib-0045] Sack, I. , Jöhrens, K. , Würfel, J. , & Braun, J. (2013). Structure‐sensitive elastography: On the viscoelastic powerlaw behavior of in vivo human tissue in health and disease. Soft Matter, 9(24), 5672–5680. 10.1039/c3sm50552a

[brb32627-bib-0046] Schmidt, J. D. , Pierce, A. F. , Guskiewicz, K. M. , Register‐Mihalik, J. K. , Pamukoff, D. N. , & Mihalik, J. P. (2016). Safe‐play knowledge, aggression, and head‐impact biomechanics in adolescent ice hockey players. Journal of Athletic Training, 51(5), 366–372. 10.4085/1062-6050-51.5.04 27111585 PMC5013701

[brb32627-bib-0047] Schwarb, H. , Johnson, C. L. , McGarry, M. D. J. , & Cohen, N. J. (2016). Medial temporal lobe viscoelasticity and relational memory performance. Neuroimage, 132, 534–541. 10.1016/j.neuroimage.2016.02.059.Medial 26931816 PMC4970644

[brb32627-bib-0048] Sherman, S. M. (2007). The thalamus is more than just a relay. Current Opinion in Neurobiology, 17(4), 417–422. 10.1016/j.conb.2007.07.003 17707635 PMC2753250

[brb32627-bib-0049] Stemper, B. D. , Shah, A. S. , Mihalik, J. P. , Harezlak, J. , Rowson, S. , Duma, S. , Riggen, L. D. , Brooks, A. , Cameron, K. L. , Giza, C. C. , Goldman, J. , Houston, M. N. , Jackson, J. , McGinty, G. , Broglio, S. P. , McAllister, T. W. , & McCrea, M. (2020). Head impact exposure in college football following a reduction in preseason practices. Medicine & Science in Sports & Exercise, 52(7), 1629–1638. 10.1249/mss.0000000000002283 32541378

[brb32627-bib-0050] Swartz, E. E. , Myers, J. L. , Cook, S. B. , Guskiewicz, K. M. , Ferrara, M. S. , Cantu, R. C. , Chang, H. , & Broglio, S. P. (2019). A helmetless‐tackling intervention in American football for decreasing head impact exposure: A randomized controlled trial. Journal of Science and Medicine in Sport, 22(10), 1102–1107. 10.1016/j.jsams.2019.05.018 31204104

[brb32627-bib-0051] Tenenbaum, G. , Stewart, E. , Singer, R. N. , & Duda, J. L. (1997). Aggression and violence in sport: An ISSP position stand. The Journal of Sports Medicine and Physical Fitness, 37, 146–150. 9239993

[brb32627-bib-0052] Van Houten, E. E. W. , Miga, M. I. , Weaver, J. B. , Kennedy, F. E. , & Paulsen, K. D. (2001). Three‐dimensional subzone‐based reconstruction algorithm for MR elastography. Magnetic Resonance in Medicine, 45(5), 827–837. 10.1002/mrm.1111 10.1002/mrm.1111 11323809

[brb32627-bib-0053] Zuckerman, M. (1982). Sensation seeking and sports. Personality and Individual Differences, 4(3), 285–292. 10.1016/0191-8869(83)90150-2

