# Supplementary material for: Relationships between aggression, sensation seeking, brain stiffness, and head impact exposure: Implications for head impact prevention in ice hockey
Source: Brain Behav. 2022 May 27;12(7):e2627. doi: 10.1002/brb3.2627 (PMC9304837; doi:10.1002/brb3.2627)
Supplement: Supplementary file 1 — Figure S1. Freesurfer segmentation of the amygdala for all participants. Figure S2: Scatterplots of significant ROI stiffness (kPa) and behavioral relationships. Figure S3. Scatterplots of significant ROI damping ratio and behavioral relationships. [file BRB3-12-e2627-s001.pdf]

Relationships between Aggression, Sensation-Seeking, Brain Stiffness, and Head Impact Exposure: Implications for Head Impact Prevention in Ice Hockey

Supplemental Information

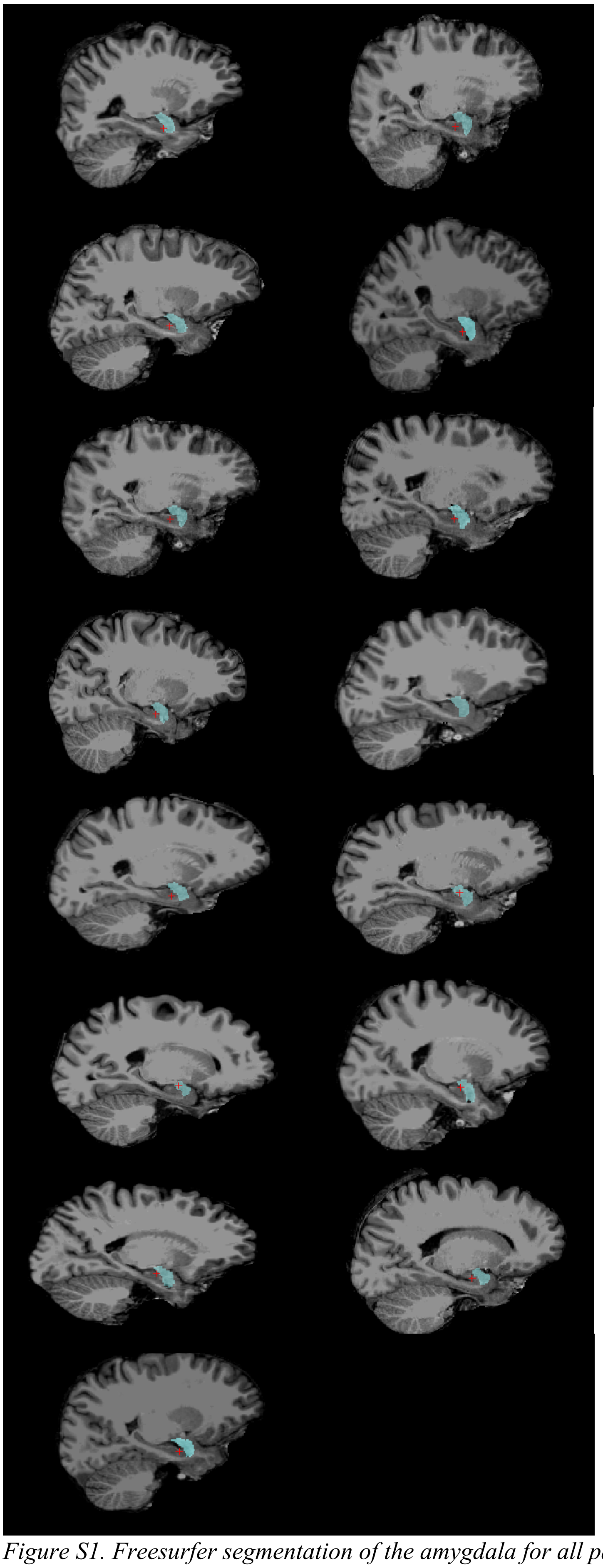

Figure S1. Freesurfer segmentation of the amygdala for all participants.

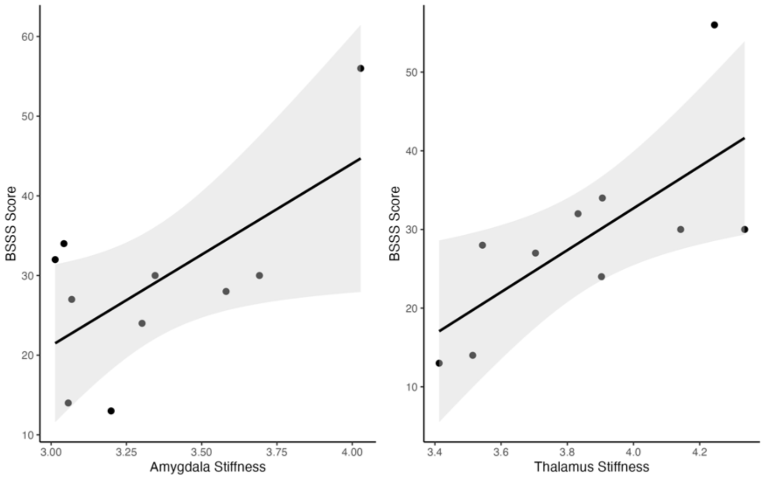

Figure S2: Scatterplots of significant ROI stiffness (kPa) and behavioral relationships.

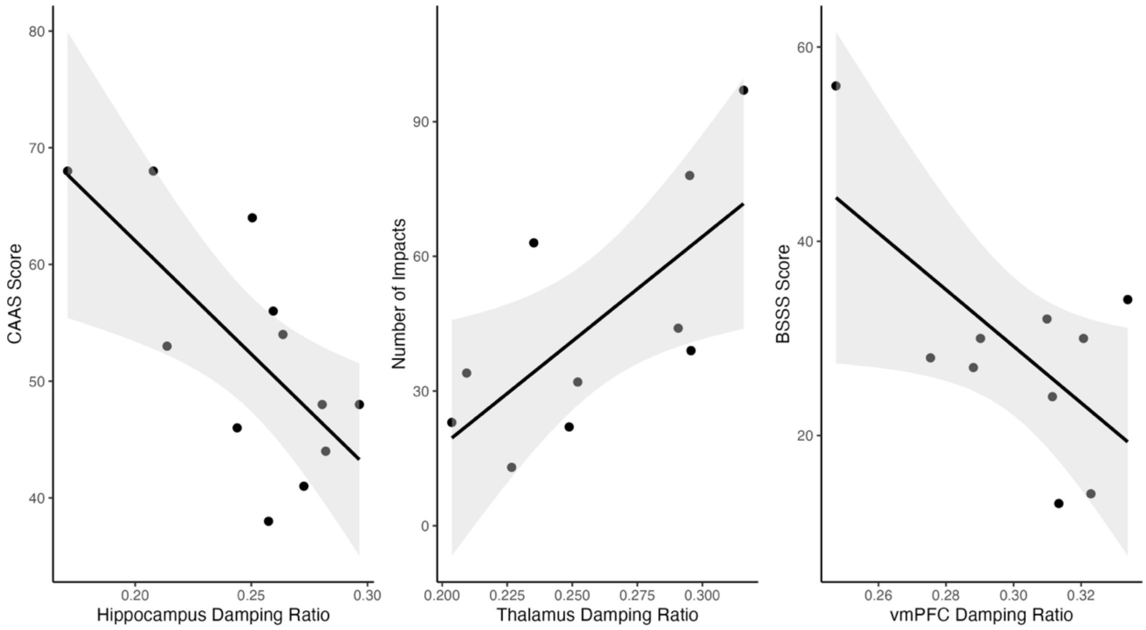

Figure S3. Scatterplots of significant ROI damping ratio and behavioral relationships.
